# A Rare Case of Dextrocardia With Atrioventricular Septal Defect and Situs Inversus in an 8‐Month‐Old Infant Complicated by Severe Acute Malnutrition

**DOI:** 10.1002/ccr3.70604

**Published:** 2025-07-30

**Authors:** Michael Tesfaye Kassa, Nuru Mohamed Ahmed, Mohamed Abdirahman Shukri, Fatumo Mohamed Abdikadir

**Affiliations:** ^1^ Department of Pediatrics Degehabur General Hospital Degehabur Ethiopia; ^2^ Department of Radiology Degehabur General Hospital Degehabur Ethiopia

**Keywords:** atrioventricular septal defect (AVSD), congenital heart disease (CHD), dextrocardia, Ethiopia, pediatric cardiology, severe acute malnutrition (SAM), situs inversus totalis (SIT)

## Abstract

The key clinical message in this case report highlights the rare coexistence of situs inversus totalis, dextrocardia, atrioventricular septal defect, and severe acute malnutrition in an Ethiopian infant. It underscores the need for heightened awareness, timely diagnosis, and integrated management strategies for such complex congenital and nutritional disorders and emphasizes the need for early surgical referral in resource‐limited settings, as delayed intervention worsens outcomes.

## 1 Introduction

Situs inversus, (rare plural: sitūs inversi) short form of the Latin “situs inversus viscerum,” is a term used to describe the inverted position of chest and abdominal organs [1].

**FIGURE 1:**
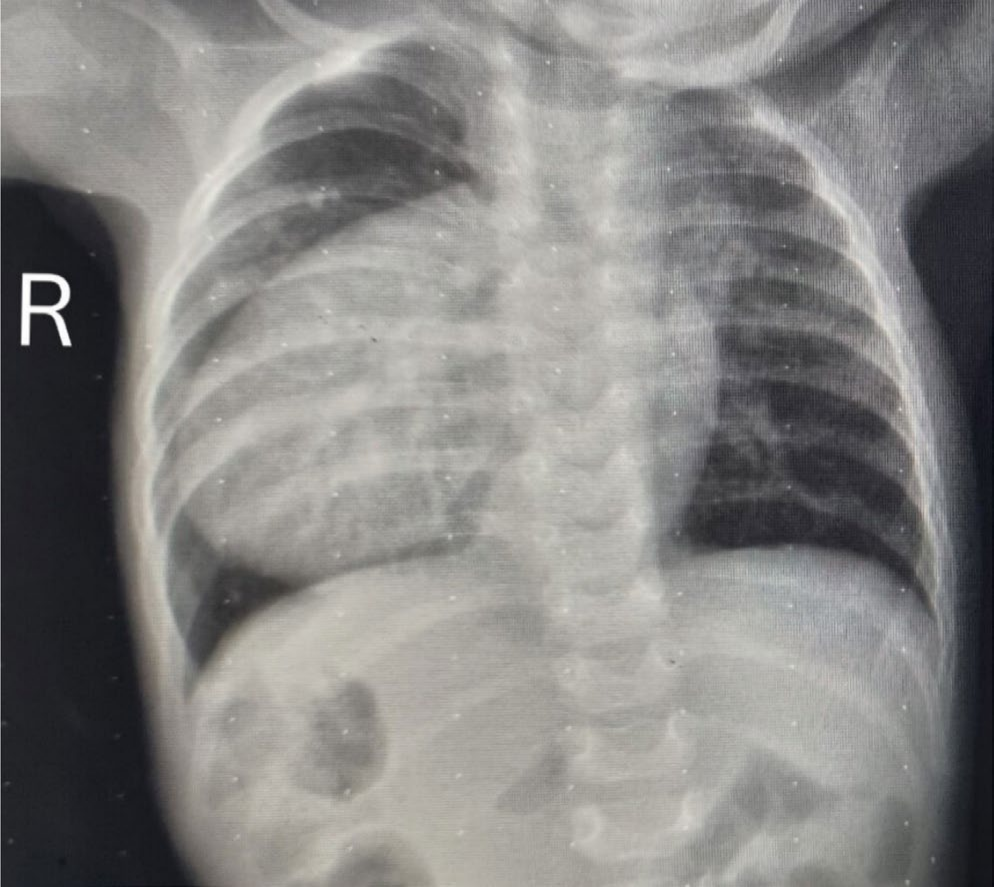
Chest X‐ray showing dextrocardia with cardiomegaly and situs inversus. Most of the heart and apex are predominantly situated in the right chest cavity, resulting in a cardiothoracic ratio of 0.63. Additionally, gastric air bubbles are seen on the right side of the abdomen.

**FIGURE 2:**
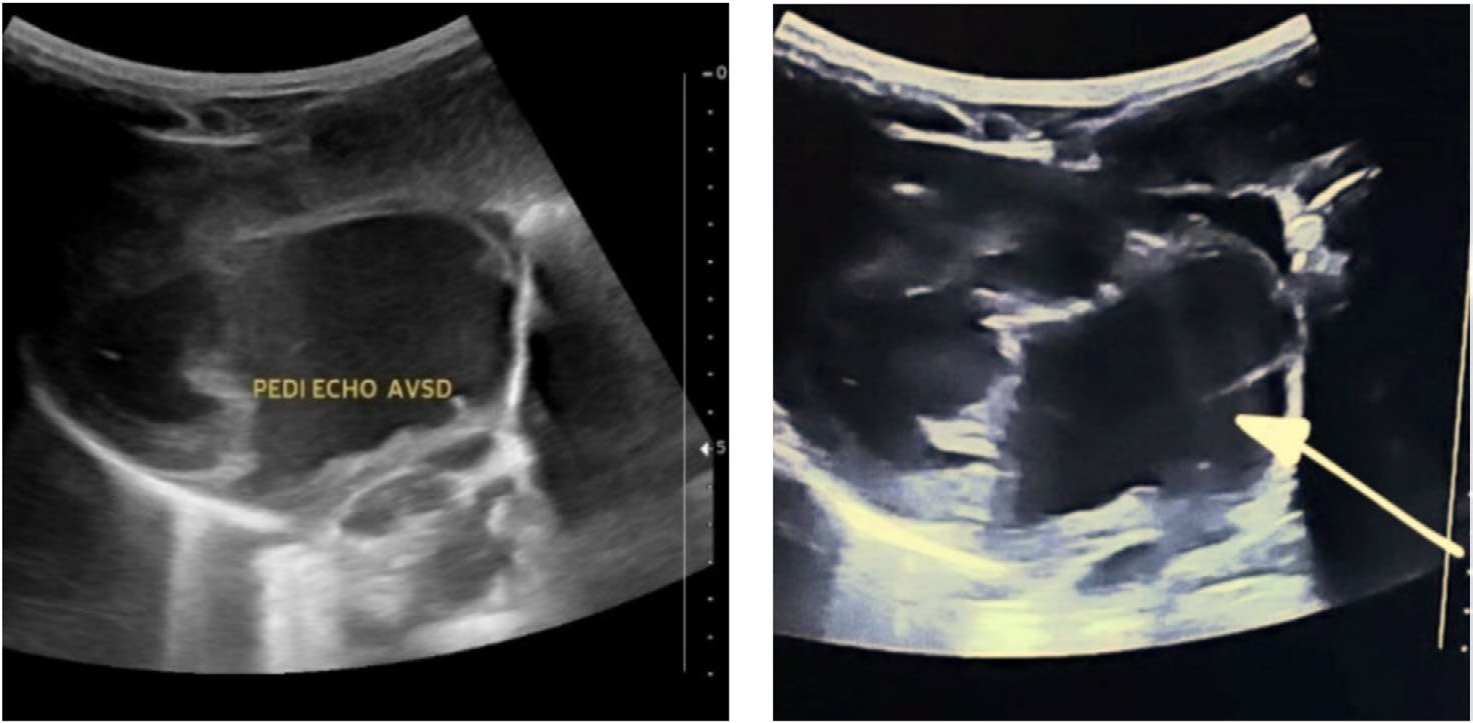
Echocardiography showing 1.5‐cm‐wide open atrioventricular septal defect involving both mitral and tricuspid valves (white arrow) insertion which shows large atrioventricular septal defect with dextrocardia and situs inversus.

**FIGURE 3:**
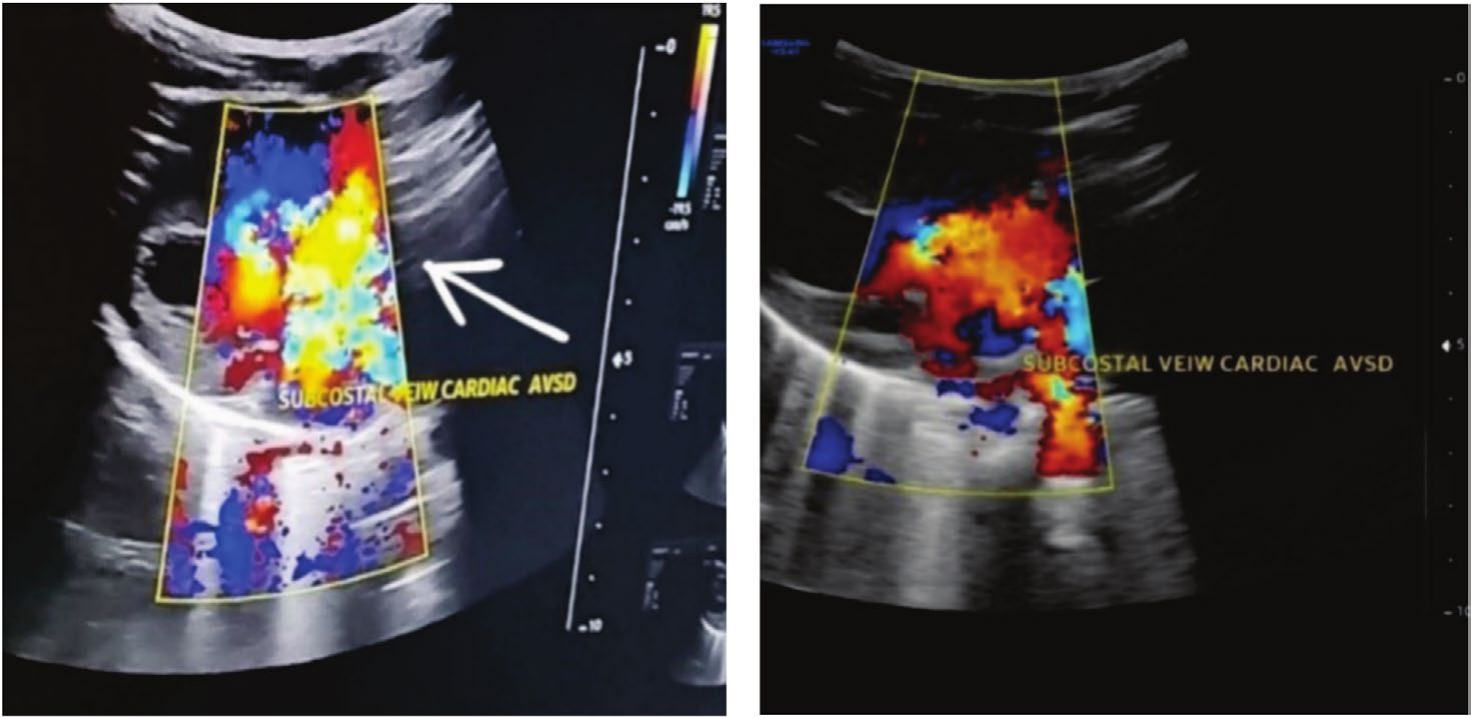
Echocardiography on subcostal view of color Doppler imaging showing a direct mixing of blood on both mitral and tricuspid valves (indicated by a white arrow), along with a 1.5‐cm‐wide open atrioventricular septal defect.

**FIGURE 4:**
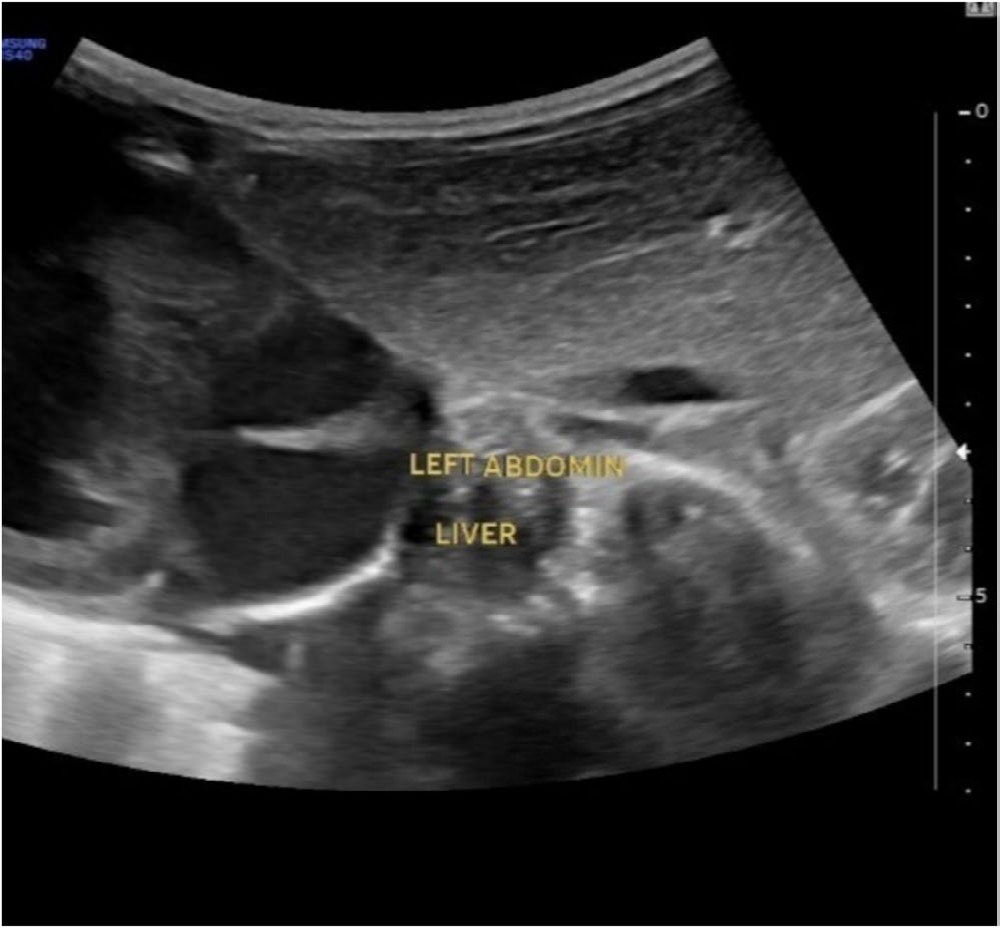
An abdominal ultrasound of the left upper quadrant reveals the liver on the left side, as opposed to its typical anatomical location.

**FIGURE 5:**
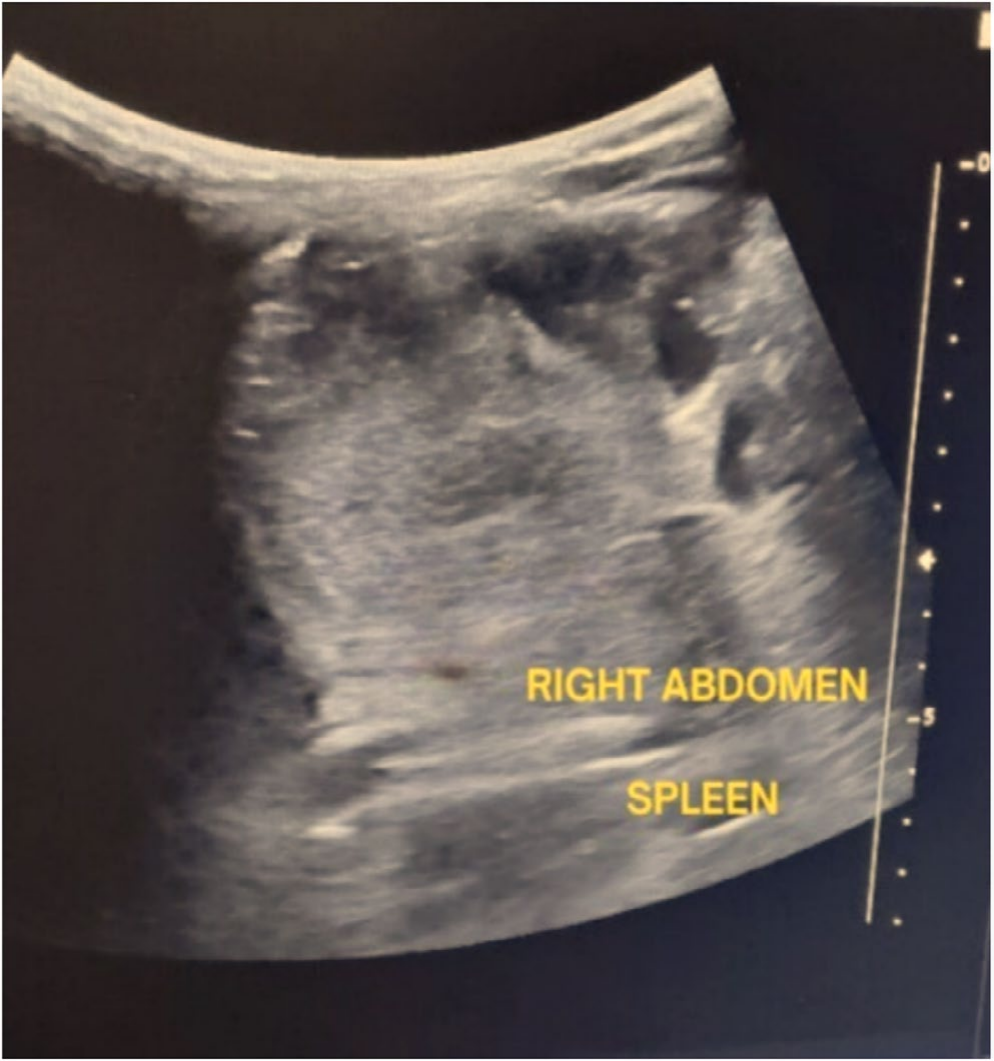
A right‐sided spleen is shown in an ultrasonography image of the right side of the abdomen as opposed to its typical anatomical location.

Situs inversus totalis is a rare congenital abnormality characterized by a mirror image transposition of both the abdominal and thoracic organs (Figures 1, 2, 3, 4, 5). While this anomaly has been known since ancient times, practicing doctors do not have much experience with it. Sidedness is regulated by genes: over 100 genes have been linked to laterality defects. The frequency of situs inversus is 1:10,000; other literature estimated the incidence of one in 10,000 to one in 50,000 patients. It is inherited by autosomal recessive traits. Gender and racial distribution are the same [2, 3].

Dextrocardia is a congenital abnormality in which the heart is positioned in the right hemithorax (Figure 1). To classify the diagnosis, the situs type must be determined. The word situs refers to the arrangement of structures within the human body, and the three types are situs solitus, situs inversus, and situs ambiguus. Situs ambiguus is the most severe and disorganized visceral misalignment disorder among the dextrocardia variants. Patients with dextrocardia with situs inversus—in which the abdominal organs are reversed or mirrored—have only a 5%–10% chance of substantial cardiac pathology [4].

Atrioventricular septal defect (AVSD) covers a spectrum of heart anomalies with a common atrioventricular connection and has an incidence of 4–5.3 per 10,000 live births (Figures 2 and 3) [5].

According to WHO, childhood severe acute malnutrition (SAM) is diagnosed when the weight‐for‐height *Z*‐score (WHZ) is < −3Z of the WHO_2006_ standards, the mid‐upper arm circumference (MUAC) is < 115 mm, there is nutritional oedema, or any combination of these parameters [6].

Malnutrition causes loss of body mass in children with congenital heart disease (CHD), especially the heart muscles. This in the long run will impair myocardial and pulmonary function of the heart. It also affects both cellular and humoral immunity with the consequent increase in the risk of recurrent infection [7].

Estimates indicate that malnutrition affects between 50% and 90% of infants with unrepaired congenital heart defects (CHD). Children with CHD, particularly those under 3 years of age and with a cyanotic lesion, tend to have lower‐than‐normal intelligence scores, which could be a result of alterations in brain weight yielding reduced cognitive functioning. Children missing this critical window of opportunity may have reduced cell numbers (including adipose, muscle, and bone cells) that continue to yield small body size and weight. Furthermore, reduced cell numbers can also translate to reduced brain tissue. In severe cases of CHD, children may experience up to a 30% reduction in brain weight [8].

Our case report aims to enhance our understanding by providing a detailed clinical description of dextrocardia and AVSD with situs inversus, along with SAM. It highlights the rarity of this condition and its clinical implications. Our case study focuses on an 8‐month‐old infant from Ethiopia, who was brought to Degehabur (Dhagaxbuur) General Hospital in the Somali region by her mother. The infant presented with symptoms of cough and rapid breathing, leading to her admission to the pediatric emergency department.

## 2 Case History and Examination

An 8‐month‐old female infant from Degehabur (Dhagaxbuur) in northeast Ethiopia was brought by her mother to the pediatric emergency department at Degehabur General Hospital. The infant presented with a 1‐month history of cough, difficulty breathing, rapid breathing, and poor sucking since 4 months of age. The mother also reported facial sweating with feeding interruptions, choking episodes during feeding, and poor weight gain since the onset of illness. There was no history of bluish discoloration of the lips or mucous membranes, diarrhea, vomiting, or contact with a person with pulmonary tuberculosis. The infant, the ninth child of the parents, was delivered at home due to limited access to nearby health facilities.

Upon examination, the patient appeared acutely ill and displayed signs of cardiorespiratory distress. Vital signs were as follows: pulse rate of 206 beats per minute, respiratory rate of 70 breaths per minute, temperature of 37.1°C, and oxygen saturation of 86% on room air, improving to 96% with intranasal oxygen at 1 L per minute. Anthropometric measurements indicated a weight of 3.9 kg and height of 58 cm, with a weight‐for‐length ratio below −3 standard deviations of the WHO *Z*‐score and a MUAC of 10.5 cm, indicative of SAM.

The head, eyes, ears, nose, and throat (HEENT) examination revealed nasal flaring and head nodding, with a normal anterior fontanel size. Chest examination showed symmetrical chest movements, intercostal and subcostal retractions, a bulged anterior chest, and normal vesicular breath sounds. Cardiovascular examination revealed the apical impulse in the right 5th intercostal space along the midclavicular line. A holosystolic murmur (Grade 4/6) was auscultated in the right 3rd–5th intercostal spaces.

## 3 Methods

Laboratory investigations revealed a hemoglobin level of 14 g/dL and a platelet count of 266 × 10^9^/mm^3^. Chest X‐ray indicated dextrocardia with situs inversus and cardiomegaly (Figure 1). Ultrasound confirmed situs inversus, where the abdominal organs are mirrored. Echocardiogram findings included dextrocardia and a large AVSD measuring 1.5 cm, affecting both the mitral and tricuspid valve insertion areas (Figure 2). Color Doppler imaging showed direct mixing of blood flow. Additionally, there was dilation in both atrial chambers and the left ventricle, while the right ventricle appeared normal in size (Figure 3).

## 4 Discussion

Situs inversus (SI) was first described in animals by Aristotle (BC. 384–322). Fabricius reported the first known case of reversal of the liver and spleen in man in 1600. A few years later, in 1650, Riolan, dean of the medical faculty of the University of Paris, reported two cases. Marco Severino first recognized dextrocardia in 1643. Küchenmeister in 1864 was the first who observed four cases in living persons by physical examination like percussion and auscultation and reported this with drawings in 1888 [2, 9].

Situs inversus is an uncommon condition in which there is transposition of the thoracic and abdominal structures. When this is associated with dextrocardia, it is termed situs inversus totalis. The typical or normal arrangement of organs, where the stomach and spleen are on the left side of the abdomen, the liver and gallbladder are on the right, and the heart is on the left side of the thorax, is referred to as *situs solitus*. Partial or complete situs inversus are both possible. Situs inversus with dextrocardia is another name for SIT. It is distinguished by having the liver on the left side of the abdomen, the stomach and spleen on the right, and the heart on the right side of the thorax. The right lung has two lobes, and the left lung has three (Figures 4 and 5) [10, 11].

This condition is present in about 0.01% of the population and is associated with CHD in 3%–5% of the cases. Dextrocardia with situs inversus has a low incidence of association with CHD, whereas isolated dextrocardia, in 90%–95% of cases, has a higher incidence of association with cardiac anomalies such as atrial or ventricular septal defect and transposition of the great vessels [3, 9].

The term “AVSD” covers a spectrum of congenital heart malformations characterized by a common atrioventricular junction coexisting with deficient atrioventricular septation. In ostium primum atrial septal defect, there are separate atrioventricular valvar orifices despite a common junction, while in complete AVSD the valve itself is also shared. The first successful surgical repair of a complete AVSD was done by Lillehei et al. in 1955. Using the technique of controlled cross‐circulation [12, 13], AVSD is relatively uncommon in the general population, with approximately 80% of all AVSD occurring in those with Down syndrome. Embryologically, these defects arise from abnormal development of the endocardial cushions, giving rise to partial, intermediate, or complete AVSD. Septal formation begins at the end of the fourth week of fetal life, when the atrioventricular endocardial cushions appear at the superior and inferior borders of the atrioventricular canal. In addition, the two lateral atrioventricular cushions appear on the right and left borders of the canal. A defect in the fusion of the superior and inferior cushions results in a persistent atrioventricular canal and thus an AVSD [14].

Acute malnutrition is a nutritional deficiency resulting from either inadequate energy or protein intake. Children with primary acute malnutrition are common in developing countries as a result of inadequate food supply caused by social, economic, and environmental factors. Secondary acute malnutrition is usually due to an underlying disease causing abnormal nutrient loss, increased energy expenditure, or decreased food intake. Acute malnutrition leads to biochemical changes based on metabolic, hormonal, and glucoregulatory mechanisms [15].

Malnutrition is a common cause of morbidity in children with CHD. Studies from developed countries have documented normalization of somatic growth when corrective surgery for CHD is performed early. In developing countries, due to resource limitations, corrective interventions for CHD are performed late, leading to a vicious cycle of congestive heart failure. Malnutrition is very common in children with CHD and is predicted by the presence of congestive heart failure, older age at correction, and lower growth potential. Corrective intervention significantly improves nutritional status on short‐term follow‐up. Inadequate intake, increased basal metabolic rate from hemodynamic changes arising from the cardiac defects, and hypoxia have all been attributed to the cause of malnutrition in these children [7, 16].

## 5 Conclusion and Results

This case report underscores the complexities and clinical implications of situs inversus totalis, dextrocardia, and AVSD in conjunction with SAM. The rarity of these conditions, particularly when occurring together, presents significant challenges in diagnosis and management. The 8‐month‐old Ethiopian infant's case highlights the urgent need for awareness and early intervention in similar patients. Addressing malnutrition is crucial, as it exacerbates the risks associated with CHD. This report aims to enhance understanding and encourage further research into effective treatment strategies for affected children.

After transferring the patient from pediatric emergency to pediatric ward, treatment was continued by putting the patient on intranasal oxygen, and Lasix and spironolactone were started, as well as ampicillin and gentamycin also initiated according to the patient's weight and as per malnutrition guideline of the country. F‐75 was also started, given by NG tube because of the increased respiratory rate to prevent the risk of aspiration until the patient's vital signs stabilized. The patient followed strictly all vital signs and clinical conditions; the patient was also followed by the WHO malnutrition follow‐up chart (multichart). After 3 days of admission in the ward, the vitals and clinical condition started to improve, and the NG tube was removed. After stabilization of the emergency condition, we planned to refer her to a higher specialized hospital for further evaluation and management.

## Author Contributions

**Michael Tesfaye Kassa:** conceptualization, data curation, formal analysis, investigation, methodology, validation, visualization, writing – original draft, writing – review and editing. **Nuru Mohamed Ahmed:** investigation. **Mohamed Abdirahman Shukri:** supervision. **Fatumo Mohamed Abdikadir:** resources.

## Ethics Statement

The report was conducted as per the Declaration of Helsinki. At the Ethiopian Somali region of Degehabur General Hospital, the hospital review board granted ethical clearance, including publishing this patient's case information. The patient's privacy and confidentiality were protected.

## Consent

The patient's mother gave written informed consent for this case report and any related images to be published.

## Conflicts of Interest

The authors declare no conflicts of interest.

## Data Availability

The datasets supporting our study have been assigned a reserved DOI in the Figshare repository (10.6084/m9.figshare.29378744) and will be made publicly available upon formal publication of our manuscript in Clinical Case Reports.
